# Topography modulates near-ground microclimate in the Mediterranean *Fagus sylvatica* treeline

**DOI:** 10.1038/s41598-021-87661-6

**Published:** 2021-04-14

**Authors:** Angelo Rita, Giuliano Bonanomi, Emilia Allevato, Marco Borghetti, Gaspare Cesarano, Valentina Mogavero, Sergio Rossi, Luigi Saulino, Maurizio Zotti, Antonio Saracino

**Affiliations:** 1 Dipartimento di Agraria, Università di Napoli Federico II, via Università 100, 80055 Portici, Italy; 2 Scuola di Scienze Agrarie, Forestali, Alimentari e Ambientali, Università della Basilicata, Viale dell’Ateneo Lucano 10, 85100 Potenza, Italy; 3 Département des Sciences Fondamentales, Université du Québec à Chicoutimi, boulevard de l’Université Chicoutimi, Quebec, Canada; 4 Key Laboratory of Vegetation Restoration and Management of Degraded Ecosystems, Guangdong Provincial Key Laboratory of Applied Botany, South China Botanical Garden, Chinese Academy of Sciences, Guangzhou, China

**Keywords:** Forest ecology, Forestry

## Abstract

Understanding processes controlling forest dynamics has become particularly important in the context of ongoing climate change, which is altering the ecological fitness and resilience of species worldwide. However, whether forest communities would be threatened by projected macroclimate change or unaffected due to the controlling effect of local site conditions is still a matter for debate. After all, forest canopy buffer climate extremes and promote microclimatic conditions, which matters for functional plant response, and act as refugia for understory species in a changing climate. Yet precisely how microclimatic conditions change in response to climate warming will depend on the extent to which vegetation structure and local topography shape air and soil temperature. In this study, we posited that forest microclimatic buffering is sensitive to local topographic conditions and canopy cover, and using meteorological stations equipped with data-loggers we measured this effect during 1 year across a climate gradient (considering aspect as a surrogate of local topography) in a Mediterranean beech treeline growing in contrasting aspects in southern Italy. During the growing season, the below-canopy near-ground temperatures were, on average, 2.4 and 1.0 °C cooler than open-field temperatures for south and north-west aspects, respectively. Overall, the temperature offset became more negative (that is, lower under-canopy temperatures at the treeline) as the open-field temperature increased, and more positive (that is, higher under-canopy temperatures at the treeline) as the open-field temperature decreased. The buffering effect was particularly evident for the treeline on the south-facing slope, where cooling of near-ground temperature was as high as 8.6 °C for the maximum temperature (in August the offset peaked at 10 °C) and as high as 2.5 °C for the average temperature. In addition, compared to the south-facing slope, the northern site exhibited less decoupling from free-air environment conditions and low variability in microclimate trends that closely track the free-air biophysical environment. Although such a decoupling effect cannot wholly isolate forest climatic conditions from macroclimate regional variability in the south-facing treeline, it has the potential to partly offset the regional macroclimatic warming experienced in the forest understory due to anthropogenic climate change.

## Introduction

Forest stands undergo spatio-temporal variations because of continuous feedbacks with local biotic and abiotic factors including disturbances. Such interactions allow exchanges of energy and matter between the canopy and the surrounding atmosphere, and play an important role in the modification of the canopy cover, stand composition, and eco-physiological behavior of trees^1^. On a global scale, there is a general consensus that macro-climatic factors such as temperature and precipitation exert a major role in controlling growth, geographical distribution as well as the health of forests. Specifically, temperature sets rather narrowly physiological growth limits to trees particularly at their altitudinal and latitudinal limit of distribution^2,3^. For instance, in a global comparison of rooting-zone temperatures at high elevation, Körner and Paulsen^4^ proposed a thermal threshold of 6.7 °C during the growing season as the thermal limitation to plant tissue formation for tree species living at the upper forest boundary. Global and regional coarse-scale climate layers such as WorldClim^5^, and CRU TS^6^ are primary predictors in the ecological biogeography to understand important implications for the functioning of forest biomes, forecast potential species range distribution and land-use variations in ongoing climate change. Empirical evidence suggests that anthropogenic climate change might drive temperate forests to critical tipping points and facilitate thermophilization of biodiversity driven by an increase of relatively warm-adapted species and loss of cold-adapted taxa^7–9^. However, whether understory forest communities would be threatened by projected macroclimate change or unaffected due to the controlling effect of local site conditions is still a matter for debate^10–12^.

To date, at the finer scale, we have a good understanding of the basic processes and mechanisms that regulate surface energy balance and temperature regimes in alpine and subalpine environments (see for instance Dozier and Outcalt^13^), and in this context empirical, correlative case studies are most useful for testing and exploring the limits of current theory, or in conjunction with observations of other processes of interest (e.g., plant population, community or ecosystem processes). Even within areas with moderate topography, ecologically significant gradients in soil moisture and near-surface air temperature can occur between slopes of different inclination and orientation, as proven by Rorison et al.^14^ who recorded a 2.5–3 °C mean annual temperature difference between north and south-facing slopes. Along roughly equivalent lines, Ackerly et al.^15^, focusing on a small montane landscape in California, assessed that thermal variability across this topographically complex area may span from 3 to 8 °C. Tree canopy also has substantial effects on rainfall interception, incoming or outgoing short-longwave solar radiation, and wind speed, resulting in a reduced lateral transfer of humidity and heat, and buffering against heat loss overnight^16–18^. Branches, leaves, and needles reflect and absorb part of the incoming solar radiation during the day, allowing less energy to reach the ground^19^. The degree of absorption is largely determined by stand age and structure, tree density, species composition, and leaf distribution^20–22^.

Interaction among the above-mentioned site-specific factors has been recognized to influence the dynamics of soil–plant–water interactions at the local scale^19,23,24^, creating a microclimate, by means of climate buffering and surrounding atmospheric decoupling over time, whose characteristics depend on either the general climate itself or the physiognomic characteristics of the vegetation cover. Such a microclimatic variability may constitute a potential important buffer in ecosystem response to macroclimatic climate change-driven variability by (i) increasing the probability of understory plants, including seedlings and regeneration of the dominant tree species, persisting locally, and (ii) providing microrefugia where understory species are capable of persisting locally despite unfavorable regional climatic conditions^2,18^.

Quantitative data about spatio-temporal relationships between the microclimate in the open areas showed the largest divergences at noon and in the early afternoon^15^. For instance, in a comparison between open-field and understory for different forest types of the Alps, Renaud et al.^25^ found the greatest thermal differences for beech and beech−silver fir forests with summer values 6 °C to 8 °C lower below the canopy compared to the open field. In a global analysis of 714 paired temperature data points, De Frenne et al.^26^ confirmed that tree canopies buffer forest floors against both maximum and minimum open-field temperatures. On average, mean and maximum understory temperatures were 1.7 °C and 4.1 °C cooler than macroclimate temperatures, respectively. Conversely, the minimum temperatures of the forest understory were 1.1 °C warmer than the temperature in the open field.

Understanding whether and to what extent the buffering capacity of forests may vary over time and across topographical features requires further insight into temperature variations over seasons, and their relations to tree phenology and stand characteristics, particularly in environments where trees grow at the edge, the treelines. In such areas, the microclimate experienced during seasons is limiting for plants when their physiological tolerance is exceeded^27,28^. Here, small fluctuations of environmental parameters may lead to changes in the specific architectural and functional components of plants (e.g. krummholz *sensu* Harsch and Bader^27^), patterns of tree establishment and mortality, which in turn alter tree spatial distribution^10,29^. In this work, we assessed how the spatio-temporal variability of macroclimate associated with canopy cover influenced the dynamics of soil surface microclimate, i.e., the near-ground temperature and soil water content. To this end, we selected the highest deciduous *Fagus sylvatica* treeline in a Mediterranean montane area (Bonanomi et al.^30,31^) as monitoring site, which represents one of the glacial refuge areas of the microthermal beech forest near to the southernmost distribution limit of the species. Our main goal was to explore the variability in near-ground microclimate imposed by contrasting topographic conditions, phenology, and canopy cover at the treeline. We described the effects of canopy cover on the near-ground air and soil temperature and soil moisture by comparing open areas and closed forest stands. We raised the hypothesis that at the treeline: (i) the forest understory is thermally buffered against macroclimatic temperatures by tree canopy cover; (ii) the influence of tree canopy on understory microclimate is greater in summer than winter; and (iii) the thermal divergence between understory and the open field follows a rather different temporal pattern according to local macroclimate and slope orientation.

## Materials and methods

### Study site

The study was conducted on Mt. Serra del Prete (39°54′ N, 16°08′ E; elevation: 2181 m a.s.l.) in Italy’s southern Apennines, close to the southern distribution limit of European beech (*Fagus sylvatica* L.). The climate of the area (data from the Campotenese meteorological station, 973 m a.s.l.) is characterized by 17.8 °C of summer temperature (average of June-July–August for 2002–2020 period) and 1.8 °C in winter (average December–January–February temperature for 2002–2020 period). The mean annual temperature is 10.1 °C and snow cover persists from November to March. Annual precipitation is 1433 mm (for 1960–1990 period), irregularly distributed throughout the year, with only 7.6% falling during the summer months. On the western mountain-side, fog and low clouds carried by perturbations from the nearby Tyrrhenian Sea mitigate soil water depletion during the dry summers. The soil originates from a fissured grayish limestone with Haplic Calcisol in the forest and shallow Rendzic Leptosol and large outcropping rocks under the grassland above the treeline^32^. The stands, dominated by single-stem and single-layer beeches, were managed until 1960 up to 1900 and 1700 m a.s.l. at the north and south aspects, respectively which indicates no anthropogenic pressure at the study sites in the last decades. Above these elevations, the regeneration strategy switches from seed to layering (i.e., the formation of adventitious roots), resulting in changes in structure from single- to multi-stem, more pronounced in the north-western than southern aspect. Starting from roughly 1500 m a.s.l. in this area the forest type switch from macrotherm, where understory vegetation covers 65% and *Sanicula europaea* L. is the dominant species, to microtherm, where understory vegetation cover ranges from 20 to 70%. Here *Adenostyles australis* (Ten.) Iamonico & Pignatti and *Galium rotundifolium* L. are the dominant species in northern and southern aspects, respectively.

### Microclimate measurements

**Figure 1:**
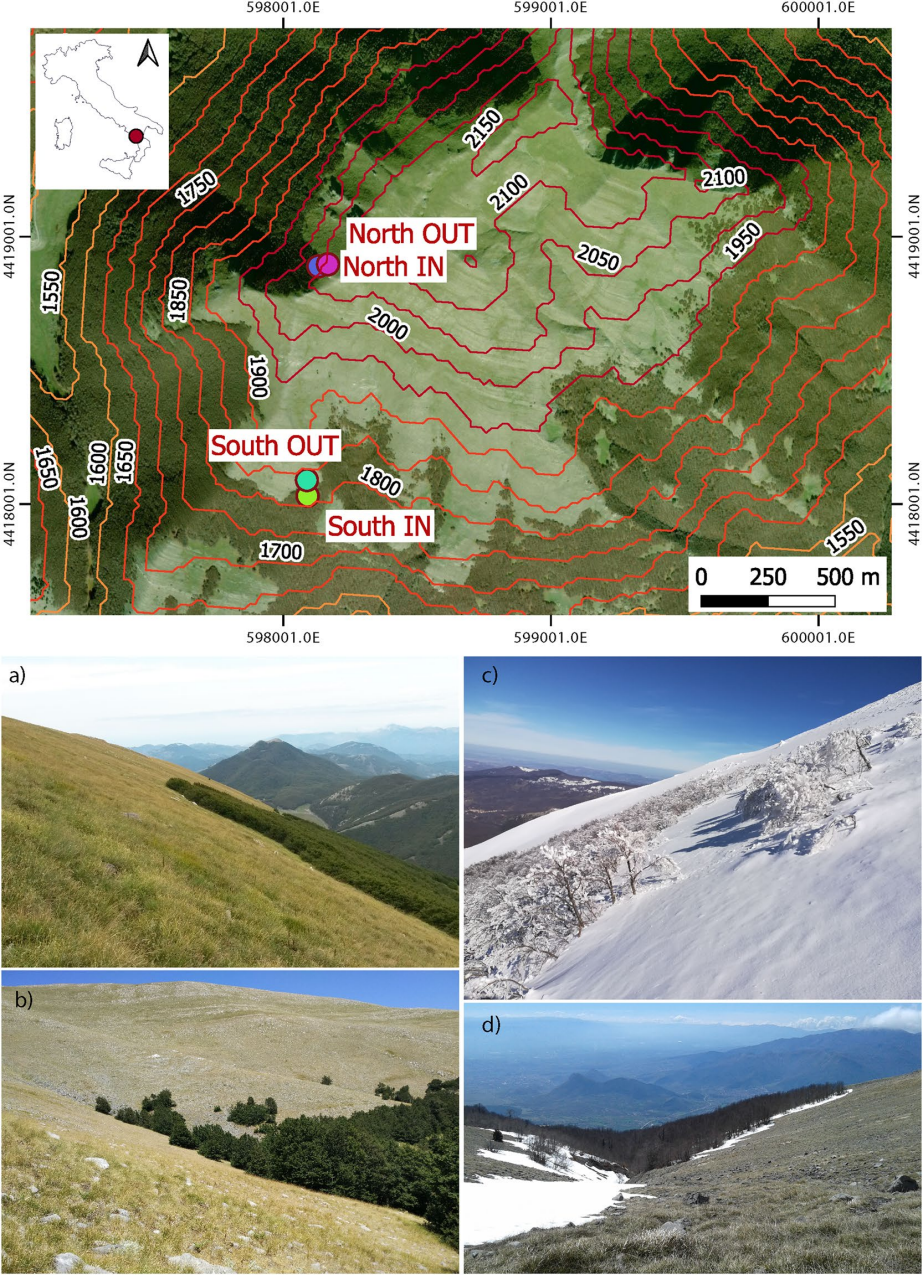
High elevation *F. sylvatica* canopy cover in the study site (Serra del Prete, 2181 m a.s.l. in the southern Apennines) with abrupt and ‘krummholz’ treeline form on south and northwest-facing slope, respectively. The markers indicate the locations of air and soil temperature and humidity sensors below-canopy (IN) and open-field (OUT) positions, on south (1840 m a.s.l) and northwest-facing slopes (2042 m a.s.l.). Contour lines represent 50 m intervals. (**a**) and (**c**) show Serra del Prete, northwest face in summer and winter, respectively. Snow depth in (**c**) was 2.5–3.0 m. (**b**) and (**d**) show Serra del Prete, south face in summer and winter, respectively. Pictures were taken by G. Bonanomi. The image on the above map is from Google Earth Pro™ (Google Inc., Mountain View, CA, USA).

Two microclimate monitoring stations were established across the uppermost treeline at two contrasting aspects (North-West vs South) and stand structures (Table 1, Fig. 1). Near-ground microclimate measurements were performed below-canopy on the north-west (2043 m a.s.l.) and south (1835 m a.s.l.) aspect. Each selected treeline has an open field near-ground microclimate measurement reference in order to document the buffering effect of the canopy cover on near-ground microclimate. Air temperature and relative humidity at 10 cm above ground and soil temperature and moisture at 10 cm depth were monitored below the canopy and in nearby grassland (open field) at 30 m distance from the from the forest edge. In each site we deployed a data logger (Em50 Decagon Devices, Inc, Pullman, WA, USA), powered by a 12 V battery replaced after 3 months, and equipped with (i) four air temperature/humidity sensor (VP-3; Decagon Devices, Inc, Pullman, WA, USA) attached to an iron pole within radiation shields to prevent direct solar insolation, which measured respectively temperature and relative humidity of air with 0.1 °C and 0.1% resolution and (ii) two integrated soil temperature and moisture sensors (5TM; Decagon Devices, Inc, Pullman, WA, USA) placed horizontally in the undisturbed mineral soil, at 10 cm depth (horizon B). Measurements were recorded every minute and averaged per half-hour from May 2016 to June 2017. Temperature records were occasionally lacking due to repeated data collection blackouts due to lightning.

**Table 1:** Summary of *F. sylvatica* treeline stand structure attributes studied in the Serra del Prete (southern Apennines).

	Northwest	South
Coordinates	39°54.874′N 16°8.826′E	39°54.377′N 16°8.840′E
Elevation (m a.s.l.)	2040	1840
Slope (°)	35.48	21.80
Azimuth (°)	112.5	0.00
Plot size (m^2^)	60	100
Stem density (n. ha^−1^)	7,651	5,500
Basal area (m^2^ ha^−1^)	50.48	10.44
Mean stand age (year)	85 (41)	43 (14)
Mean diameter at 1.30 m (cm)	8.65 (4.50)	4.65 (1.75)
Mean total height (m)	5.13 (3.01)	7.36 (2.34)
Mean live crown base height (m)	0.93 (0.20)	2.58 (0.83)
Mean height-to-live crown (m)	5.01 (2.81)	4.79 (1.53)
Mean stem form index (–)	1.58 (0.23)	1.11 (0.06)
Plant Area Index	4.78 (0.29)	3.68 (0.35)
SOS	130 ± 12	120 ± 12
EOS	310 ± 13	340 ± 9.3
LOS	200 ± 2.3	220 ± 14

In brackets values of 1st standard deviation are reported. Metrics of land surface phenology and greenness are derived from NDVI time series extracted from MOD13Q1 250 m spatial resolution and 16-day temporal resolution for the period 2016–2019. SOS, start of the growing season; EOS, end of the growing season; LOS, length of the growing season (see supplementary method for details of the numerical calculation).

### Stand attributes

Stand attributes at the micrometeorological recorded sites were measured in transects at least 6 m long × 10 m wide parallel to isohypse and calibrated to account for the slope on both treelines (Table 1). Diameter at breast height, stem form index (i.e., stem length:stem height ratio in the first 2 m from the ground), total and live crown base heights were measured in all trees of the transect. Plant area index (PAI) was measured in summer during the fully leafed period by using the LAI 2000 Plant Canopy Analyzer (Li-Cor, Lincoln, NE, USA).

### Climatic data analysis

We calculated the mean and the standard deviation (SD) on the long-term raw data to examine the variation of the data and detect statistical outliers. Values exceeding the range of mean ± 3 SD were considered as potential outliers and were then inspected manually and excluded from the subsequent analysis if they did occur. Data were also aggregated to daily maximum and minimum air and soil temperatures, and soil moisture.

Differences in the daily mean of near-ground air and soil temperature among selected sites were first tested via ANOVA analysis followed by Tukey's post hoc multiple comparisons tests (P < 0.05). Then we carried out a multilevel-modeling framework using intercept-only linear mixed-effects models (LMMs) without fixed predictor variables but using nested month, day and hour as a random intercept term to account for random variability. The intercept of intercept-only models represents the average magnitude of the temperature offset (i.e., values calculated as the temperatures inside forests minus macroclimate temperatures in the open field) of forests in summer (June–July–August), winter (December–January–February), and through the growing season (from May to September) while accounting for the non-independence among replicates from the same measurement. When fitting our intercept-only LMMs, we used the restricted maximum likelihood method in the *lmer* function from the *lme4*^33^ package in R^34^ version 3.6.2.

We tested for nonlinearity of the relationships between the average temperatures outside the forest and the forest temperature offset (i.e., the difference between the below-canopy and open-field temperature) during the growing season using General Additive Mixed Models (GAMMs) for both stands including open-field temperatures and daytime (as sin and cos of hours) as fixed effects and month-day as random effects with the *mgcv*^35^ package. We assessed to what extent open-field temperatures predicted variation in the forest floor temperature during the growing season and major differences between the two contrasting treelines were computed in terms of their buffering capacity of the canopy cover between northwest and south-facing slopes. Indeed, as well explained by Davis et al.^36^, this metric is able to capture the combined effect of the decoupling and buffering processes. We then fitted LMMs with open-field temperatures and daytime (as sin and cos of hours) as fixed effects, and month and day as a nested random effect using restricted maximum likelihood in the *lmer* function from the *lme4* package. We also performed the χ^2^ test by comparing the univariate LMM, including a single predictor with the baseline intercept-only model. Goodness-of-fit was determined by calculating the marginal and conditional coefficient of determination (R^2^) as previously reported using the ‘r.*squared GLMM’* function in the *MuMIn*^37^ package.

## Results

### Below-canopy vs. open-field near-ground air temperature

**Figure 2:**
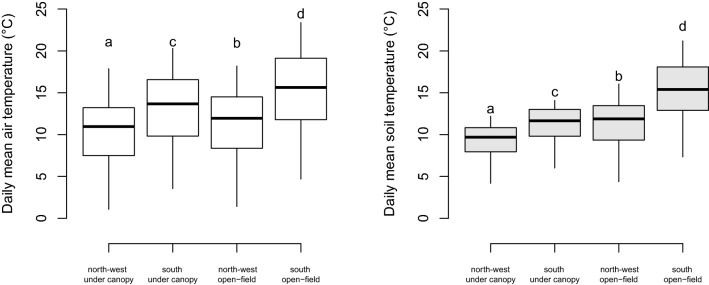
Temperatures categorized by their position at the treeline. Daily mean air (left) and soil (right) temperature for the growing season (from May to September). Each box shows the 75th to 25th percentiles, and the line inside represents the median; upper and lower marks are the largest to smallest observation values, which are less than or equal to the upper and lower quartile plus 1.5 times the length of the interquartile range; circles outside the lower–upper mark range are outliers. Different letters indicate significant differences for P < 0.05.

During the monitored period, the minimum temperature recorded was − 10.1 °C on the south-facing slope below the canopy at 2:00 a.m. on November 30th 2016, and − 9.8 at 0:30 a.m. on January 8th 2017 on the NW open field; the maximum air temperature was 39.45 °C at 3:30 p.m. on July 12th 2016 (south-facing, open-field). The greatest temperature variability was observed in April and May, when the temperature experienced high daily fluctuations (Table S1, Supplementary Figs. S1 and S2). Overall, the air and soil temperatures were considerably higher on the south than on the northwest slope. During the period from May to September, on the north-facing slope, the daily mean air temperature was 10.4 °C and 11.5 °C for below canopy and open field, respectively. On the south-facing slope, the daily mean air temperature was 13.1 °C and 15.3 °C for below canopy and open field, respectively. Significant differences were also detected for the soil average daily temperature which was 11.1 °C and 10.4 °C for the northwest aspect, and 15.1 °C and 13.7 °C for the south, for below canopy and open field, respectively (Fig. 2, see also Table S1).

**Figure 3:**
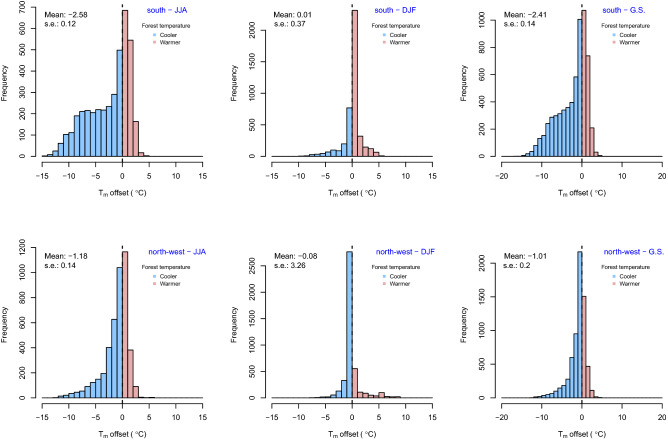
Temperature offset values for average mean temperatures during summer (JJA), winter (DJF), and the growing season (G.S., i.e., from May to September). Mean temperatures are consistently cooler under-canopy (microclimate) compared to open field (macroclimate). Temperature offsets (mean ± s.e.) are based on mixed-effects models with months, days, and hours as nested random-effect terms (the full statistics are reported in Supplementary Material Table S2). Y-axis scales are not fixed.

**Figure 4:**
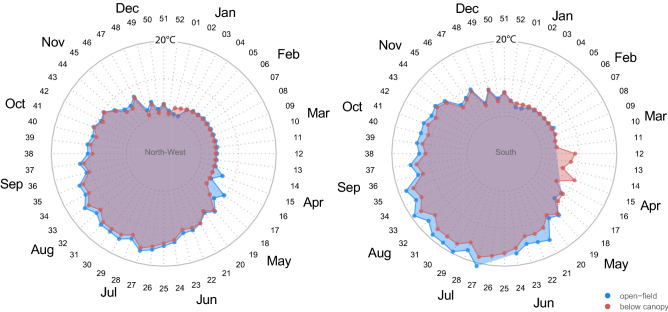
Average weekly near-ground air temperature recorded in the open field (blue) and below the forest canopy (red) on North-West (left) and South (right) aspects. Black numbers in the radar charts are the week number while gray numbers are the temperature (in °C).

Overall, daily and weekly patterns indicated that canopies buffer forest floors against average (Figs. 3 and 4), maximum, and minimum open-field (Supplementary Figs. S3 and S4) temperatures. In summer, the below-canopy temperatures were, on average, 2.58 ± 0.12 °C and 1.18 ± 0.14 °C cooler than those in the open field for south and northwest aspects, respectively (mean ± s.e., mixed-effects models: both *p* < 0.001). This is particularly evident in August for the treeline on the south-facing slope, where a maximum temperature difference of about 10 °C (raw measurements from minute-interval) between open-field and below-canopy is recorded. No significant offset was found for the average temperature in winter (DJF) for south and northwest aspects (mixed-effects model: not significant; Supplementary Table S2). During the growing season (i.e., from May to September), the below-canopy temperature was, on average, 2.41 ± 0.14 °C and 1.01 ± 0.20 °C cooler than that of the open field for south and northwest aspects, respectively (Fig. 3; mixed-effects models, *p* < 0.001). Maximum temperatures in summer were 8.63 ± 0.4 °C and 5.42 ± 0.81 °C cooler under-canopy than open-field for south and northwest aspects, respectively (mixed-effects models: both *p* < 0.001, Supplementary Fig. S3). Conversely, the below-canopy minimum temperature in summer was 0.90 ± 0.17 °C and 0.31 ± 0.14 °C warmer than that of the open field for south- and northwest-facing slopes, respectively (Supplementary Fig. S4).

An uncoupled spatio-temporal pattern of temperatures between sampled sites was common in our dataset, mostly during spring. For instance, on the northwest-facing treeline, a large offset (more than 5 °C) was found in the average temperature in April (between the 15th and 16th week of the year) between under-canopy and open-field conditions (Fig. 4). At this time, the snowpack under the canopy on the northwest-facing slope persists compared to the adjacent open-field where it has already melted due to the increase in seasonal temperatures (see Fig. 1d for an example of below-canopy snow persistence and Supplementary Fig. S7). The buffering effect of the forest canopy against the average temperature is recorded in almost all the periods of the year, especially in the central hours of the day when open-field temperatures peaked (Supplementary Fig. S5).

**Figure 5:**
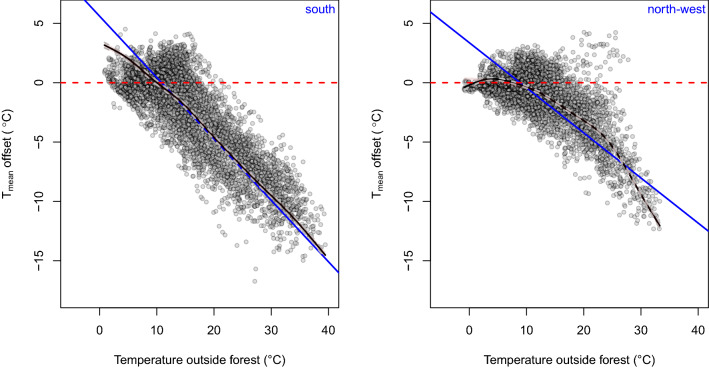
Relationships between the average near-ground temperatures in open-field (set as reference) and below-canopy temperature offset during the growing season (from May to September) using General Additive Mixed Models (GAMMs) for south (left panel) and northwest (right panel) treelines, respectively. Solid black lines show the fitted GAMMs with month, day and hour as nested random-effect terms (dashed gray lines and gray shaded areas between the dashed lines show the standard error around the predicted values); solid blue lines show the fitted linear mixed-effects models (LMMs) with a nested random-effect term for South (intercept = 5.6, slope = -0.51; p < 0.001) and North (intercept = 3.37, slope = -0.37; *p* < 0.001); red dashed lines show the null line (temperature offset = 0 °C or below-canopy equals open-field temperature). G.S. represents the growing season from May to September.

During the growing season, the relationship between temperature offset and open-field temperature is linear for the southern stand (Fig. 5 and Supplementary Table S3), while it deviates from linearity for the northern stand at temperatures < 10° and > 27 °C. The slope of the fitted regression lines, computed by means of the mixed-effects models, was steeper for the treeline facing south (slope = − 0.51) compared to northwest (slope = − 0.37).

**Figure 6:**
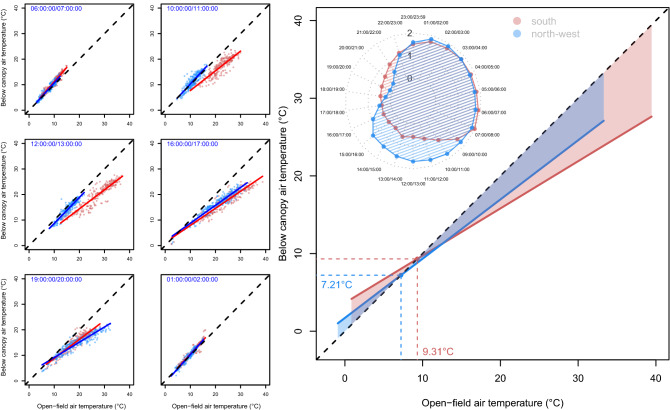
Relationship between near-ground open-field (set as reference) and below-canopy daily mean temperature of the growing season (from May to September). Blue and red circles represent the mean daily temperature of northwestern and southern treelines, respectively. The full line represents the linear regression fit (northwestern, blue line: intercept = 1.73, slope = 0.76; southern, red line: intercept = 3.65, slope = 0.60). The dashed black line represents the 1:1 relationship (temperature offset = 0 °C or below-canopy equals open-field temperature). Positive values (shaded pink area) indicate overall near-ground warmer conditions under the canopy than at the reference site, while negative values (shaded blue area) indicate cooler air under the canopy than reference conditions. Radar inset plot shows the hourly slope of the fitted lines. Full statistics are reported in Supplementary Material Table S4.

The linear mixed model between below-canopy temperatures and the open-field temperatures during the growing season summarizes the microclimatic buffering effect of the forest canopies at the treeline. The slope of fitted lines measure below-canopy microclimate variability and can represent the strength and magnitude of ‘decoupling’ (i.e., when site microclimate is isolated from macroclimatic conditions) when the reference sensor represents open-field conditions (Fig. 6). At contrasting aspects, canopies showed substantial differences (*p*-value < 0.001, ANCOVA with the aspect as dummy) in their ‘buffering capacity’ i.e., the cumulative area between the fitted regression lines in Fig. 6 (sensu Davis et al.^36^) where the shaded areas below the 1:1 line indicate cooler in forest compared to the open-field conditions, and vice-versa. Figure 6 shows that the near-ground temperature under the canopy increases when the open field warms up, but at a lower rate at the southern treeline (slope coefficient of 0.60) compared to the northwest-facing treeline (slope coefficient of 0.76). For instance, at the southern treeline the below-canopy near-ground temperature may be either higher or lower than at the northern treeline, depending on the reference temperature. At left side of the crossing point in Fig. 6 (i.e., open-field air temperatures lower than the below canopy air temperature) the buffering capacity, quantified on the summed area (thermal sum via integration) between the fitted 1:1 lines, of the southern treeline during the growing season (i.e., from May to September) is 13.7 °C when open-field temperatures range from 0.85 to 9.31 °C, while for the northwestern treeline is 7.74 °C when open-field temperatures range from − 0.91 to 7.21 °C. When the when open-field temperature is warmer than the below canopy air temperature the buffering capacity of the southern treeline is 138.9 °C when open-field temperatures range from 9.31 to 39.45 °C, while for the northwestern treeline is 82.1 °C when open-field temperatures range from 7.21 to 33.29 °C. The pattern of decoupling shows that both the understory average temperature at the northwest and south aspects is coupled with the open-field temperature from 23:00 p.m. to 7:00 a.m. From 10:00 a.m. to 13:00 p.m. the average temperature at the south aspect decoupled with the open-field temperature, while at the northwest aspect the temperature is still coupled. In the afternoon till sunset (from 16:00 p.m. to 21:00 p.m.), both the below-canopy average temperature at the northwestern and southern aspects are decoupled from the open-field temperature (Fig. 6).

### Below-canopy vs. open-field soil temperature and moisture

**Figure 7:**
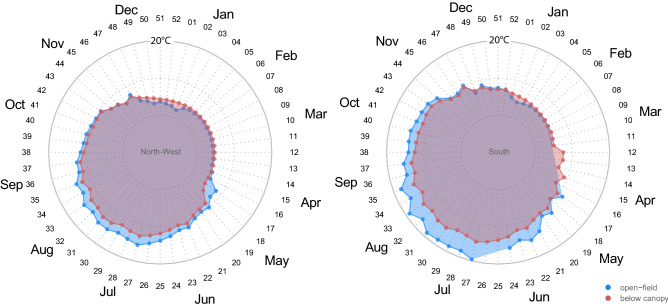
Average weekly soil temperature recorded in the open field (blue) and below the forest canopy (red) at the North-West (left) and South (right) aspect. Black numbers in the radar charts are the week number from the start of the year while gray numbers indicate the temperature (in °C).

The average temperature of the soil showed less weekly variability than the average air temperature (Fig. 7, and Supplementary Fig. S6 for average daily soil temperature). However, the temperature offset between the understory and open-field conditions was much higher than the near-ground air temperature. The above difference was particularly high in the summer months when the southern aspect was on average 13 °C. By contrast, in winter, the offset between temperatures is positive, meaning warmer temperatures below the canopy.

**Figure 8:**
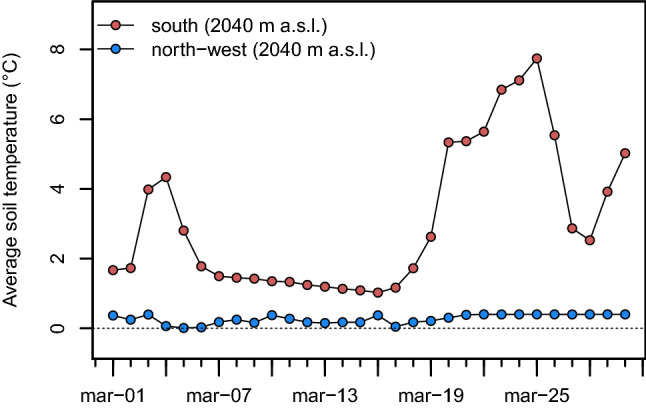
Comparison of average soil near-ground temperature in the open field during March 2017 on north-west (blue) and south-facing slopes (red).

**Figure 9:**
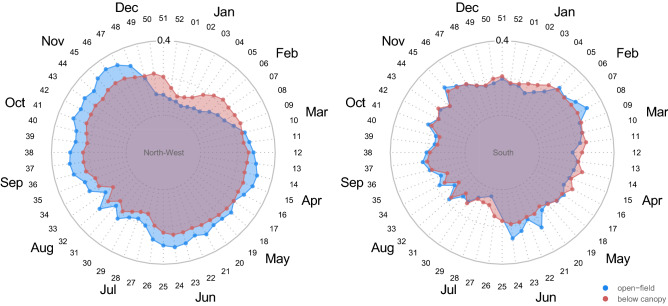
Average weekly soil near-ground moisture recorded in the open field (blue) and below the forest canopy (red) at the North-West (left) and South (right) aspects. Black numbers in the radar charts are the week number from the start of the year while gray numbers represent the soil moisture scale (m^3^/m^3^).

A difference between the temperatures recorded at comparable elevations under open-field conditions on the northwest- and south-facing slopes (where we deployed further instrumental measurement at 2040 m. a.s.l. on the southern slope) in March. At this time, the presence of the snow cover (not yet melted in this month, Supplementary Fig. S7) on the NW aspect keeps the soil temperature close to 0 °C, whereas on the southern slope, where periods of snow and non-snow cover alternate, the temperature range occurring in this month makes average soil temperatures fluctuate considerably (Fig. 8). On the south-facing slope this caused a large thermal amplitude (up to 8 °C). The NW treeline shows almost throughout the year a contrasting soil moisture pattern, which is higher on the open field, in the prairie, compared to the understory (Fig. 9, and Supplementary Fig. S8 for average daily soil moisture). Conversely, in the months of December, January, and February the moisture content was higher in the open field compared to the covered soil.

## Discussion

Our results provide substantial evidence that local modification of the climate by topography and canopy cover creates microclimates at the near-ground forest surface which can partly outweigh regional macroclimate variability. Major microclimatic differences between south- and north-west-facing treelines are attributed primarily to the difference in the insolation-radiation balance on the two slopes (i.e., southern aspects can receive up to six times more solar radiation than northern aspects in the middle hours of the day, Supplementary Fig. S9), which produces marked effects on the temperature and moisture of the soil and air on the two slopes^38^.

Beyond differences imposed by the slope exposure itself, canopy cover also marks the seasonal pattern of treeline microclimate by playing a key role in regulating the documented offset of average and maximum summer temperatures. That is, the Mediterranean beech treeline provides a highly heterogeneous thermal environment, where the under-canopy is not only cooler on average than surrounding open fields, but negative maximum temperature offset (cooler in the forest) and positive minimum temperature offset (warmer in the forest) also entail lower temperature variability below the canopy on both aspects, as also observed by Renaud et al.^25^ in the case of alpine forests in Switzerland and by Bader et al.^39^ for the tropical alpine tree lines in the Andes.

At the study sites the average air temperature offset that we documented is consistent with the general patterns observed in temperate regions across the globe (ranging from 1.5 to 5 °C), spanning different forest types and structures^26,40,41^. These above-mentioned differences in temperature are biologically significant, as experimental studies have shown that high air temperature (and hence air VPD), regardless of its primary mechanism, directly affects whole-plant growth and reproduction as well as promotes soil microbial activity and nitrogen mineralization^42,43^. For instance, Keitel et al.^44^ found a strong link between stomatal conductance and air temperature, resulting in a relationship between air temperature and transpiration of beeches. Analyzing the dynamics of radial stem variance and radial growth, van der Maaten et al.^45^ observed that daily mean and maximum air temperatures and soil water content were found to explain 59% of the variance in the day-to-day stem radius of beech. Compared to open-field, understory vegetation also benefits from the smoothed temperature from overstory, which affects the growing conditions and the carbon (C) uptake linked to an efficient trade-off between photosynthesis and transpiration. Besides, vegetation-mediated changes in microclimate have been observed e.g. by Smith and Johnson^46^ to reduce annual woodland soil respiration relative to grassland (by ∼ 38%), resulting in a greatly slowed soil C cycling in woodlands with an estimated C turnover rate of 15 years slower than grasslands. Taken together, the above-cited studies yield insights into the role of microclimate temperature on growth and carbon cycles of temperate beech forests (but see Wu et al.^47^).

At the north-western treeline, the air temperature offset is much lower than at the southern treeline (an understory-open-field difference of about 1 °C), intuitively because of the lower daytime temperature range. This offset became more negative (that is, lower under-canopy temperatures at the treeline) as the open-field temperature increased, and more positive (that is, higher under-canopy temperatures at the treeline) as the open-site temperature decreased. In this context, large differences emerged in the relationship between temperature offset and macroclimate between NW and S-facing slopes i.e., for 1 °C macroclimate warming/cooling (from the mean temperature value) the maximum temperature difference in microclimate warming/cooling between southern and north-western treelines increased. Roughly speaking, the forest canopy treeline on the south-facing slope has a substantial cooling effect in summer and heating in winter, compared to the northwest slope. The results supported by this study closely agree with empirical research showing the existence of a more pronounced thermal gradient on south or southwest-facing edges^48,49^, albeit with some exceptions (e.g., Treml and Banaš^50^). It is worth noting that the maximum temperature offset values recorded for the growing season between below-canopy versus open-field temperature exceed 15 °C for the south-facing beech treeline. This very high value far exceeds the values for other forest types across biomes reported by De Frenne et al.^26^, testifying to the high buffering capacity of Mediterranean beech stands under such latitudinal and topographical conditions. Furthermore, for the north-western treeline we cannot exclude that the fair non-linearity of the relationships between the average near-ground extreme high/low open-field temperatures and below-canopy temperature offset during the growing season may be attributed to the effect of plant stature and canopy structure (see Table 1) affecting a nonlinear light absorption along the vertical canopy profile^51^. Indeed, the higher plant area index (PAI) coupled with the aerodynamic crowns closer arranged to the ground at the north-western than southern treeline, is likely to have played a crucial role in reducing incoming solar radiation to the ground and hence smoothing temperatures, as documented for instance by Frey et al.^52^. This would not minimize the role of landscape topography or other background environmental constraints for the forest microclimate at high elevation, but we acknowledge that the microscale variation in canopy cover, coupled with the unbalanced microclimate measurements—which ignores possible within-site spatial variation in near-surface air temperatures—might be underestimated in our results^53,54^.

The rather peculiar pattern of decoupling of below-canopy temperature vs. open-field suggests that the south-facing beech treeline was likely to be more decoupled from the regional free air conditions than the north-western beech treeline, particularly during full sunshine hours. This result might sound rather counterintuitive since the lower stature and height above ground of the northern exposed trees should benefit from a strong aerodynamic decoupling to the free atmosphere, as outlined above. However, we point out that this is consistent either with the difference in the solar radiation balance on opposite aspects or with the difference in altitude between treelines, where the natural disturbances coupled with human influences are the most obvious reasons for the southern treeline position below the target elevation^30^.

Although such a decoupling effect cannot completely isolate forest climatic conditions from macroclimate fluctuations, it has the potential to partly offset the regional macroclimatic warming experienced in the forest understory due to anthropogenic climate change^7^. As such, closed forest canopies might provide a line of defense against the impacts of current and future warming on the ecological processes that influence high elevation Mediterranean forest ecosystems^7,29^ (for example, regeneration, demography and community reshuffling, litter decomposition, and soil water and nutrient cycling, among the others). This vegetation–microclimate feedbacks would then have major implications for the ability of Mediterranean beech at the subalpine belt to (i) (self-)sustain and facilitate tree regeneration because, compared with the adjacent grassland areas, it significantly reduces the probability of freezing‐induced damages in seedlings growing beneath shelter tree canopies^55,56^; (ii) improve the stability of the ‘cooler’ soil C pool via lower levels of heterotrophic respiration in woodland, which in turn affects C turnover^57^; and (iii) act as microrefugia for specific understory cold-adapted taxa against a rapid thermophilization of temperate forests that affects floristic turnover in favor of relatively warm-adapted species^8,14^. To this, De Frenne et al.^8^ reported that the significant understory community turnover of one-third of the species present across temperate regions was consistent at higher altitudes with the warming climate (estimated thermophilization rate 0.041 °C decade^−1^), which led to novel species assemblages. However, in the Mediterranean bio-climate, the mechanism by which southern exposures might support microrefugia is still not well addressed since temperature effects due to aspect must also be balanced against water loss at such sites.

As offsetting was strongest for maximum temperatures, we might expect also extreme events in the Mediterranean basin such as heat waves to be more strongly attenuated than gradual temperature changes. Canopy-air temperature interaction phenomena also become particularly important during late spring frosts, which may severely affect the photochemical efficiency of young leaves, thereby influencing development of seedlings and shoots for *F. sylvatica*^58,59^.

Our results showed that soil temperature is also controlled by canopy cover as previously detailed for the treeline ecotone in Nepal^60^. The denser the canopy cover (i.e., north-western treeline stand, see Table 1), the smaller the difference between below-canopy and open-field soil temperatures. Albeit with less daytime variability, our results show that the average soil temperature is strongly coupled with the air temperature (see Supplementary Tables S5 and S6), with a strong cooling effect provided by the canopy in summer. Consistent with our findings, this effect has been empirically estimated to be about 4–5 °C (difference compared to the open field) also for grassland communities in summer up to 80–100 cm soil depth by Liechty et al.^61^. On the south-facing slope, the amplitude of the average daily soil temperature was subject to huge variations (up to 8 °C) as a consequence of frequent snowmelt cycles. Here snowpack becomes isothermal earlier in the spring when radiation differences between slope facets are highly dependent on solar angles, thus increasing the role of aspect differences between sites. The negative direct/indirect consequences would be a dramatic effect on the growth rate (e.g., Peterson and Peterson^62^) or frost-induced earlier growing-season desiccation which may have a crucial effect on seedling establishment, mostly at the initial life stage.

At our treelines, the soil moisture showed high spatio-temporal variability, affected either directly or indirectly by both precipitation and temperature feedbacks. Such drying–rewetting cycles of soils would affect not only the microbial biomass and the mineralization of soil organic carbon (i.e., the “Birch effect” in Jarvis et al.^63^) but would also impose several constraints on seedling survival, mostly at their initial life stage when shallow root systems and reduced soil water availability are critical factors for their performance under common seasonal droughts. At southern exposed treelines the soil moisture pattern between open-field and below-canopy conditions was in phase during most of the growing season suggested that the soil moisture is modulated by the atmosphere VPD rather than by the different physiognomy of the cover. We argue that the peculiar pattern of soil moisture at the north-western treeline (i.e., higher in the open field compared to below-canopy in nonwinter months) may have three non-mutually exclusive explanations. First, the north-western treeline stand with a dense canopy would intercept and transpire more water because of the coupling with the macroclimate and therefore deplete soil moisture faster than areas with a sparse canopy or none at all^19^. Throughfall can be also intercepted and retained by the well-packed undecomposed litter layer decreasing the percolation into the upper soil layer. Additionally, this could be attributed to the different organic characteristics of soils: in the open field, the prairie soil appears more soaked compared to the forest soil where the humus layers, characteristic of understory beech forest, absorb from two to four times their weight of water^64^.

## Conclusion

This study compared the below-canopy microclimate to the local climate in order to explore the variability in the near-ground microclimate imposed by contrasting topographic conditions, phenology, and canopy cover in the highest deciduous *Fagus sylvatica* Mediterranean treeline. Our findings provide strong support for the notion that local landscape structure, as delineated by topographic exposure, and canopy cover create microclimates at the forest near-ground, which partly outweigh macroclimate variability. In particular, we found that (i) during the growing season, below-canopy near-ground temperatures were, on average, cooler than those in the open field on both aspects (S and NW) investigated, and (ii) compared to the south-facing slope, the northern site exhibited less decoupling from free-air environment conditions and low variability in microclimate trends that closely track the free-air biophysical environment. The unbalanced microclimate measurements available for this study, coupled with the high heterogeneous environment, seem to preclude a comprehensive assessment of the potential effects of ongoing climate change on the local microclimate condition at the monitoring site. However, the results outlined above provide evidence that topographically heterogeneous subalpine areas are more decoupled, and thus more climatically stable, than open fields. This would lead not only to cooler and milder local understory conditions than open-field conditions during summer/day- and winter/night-time, but also, most importantly, to weak coupling between understory and regional open-field inter-annual climatic fluctuations. Our results suggest that current microclimates have the potential to offset climate warming at the local scale and reduce the disequilibrium between ecosystem responses and anthropogenic climate change by (i) facilitating forest natural regeneration at the subalpine belt, (ii) promoting the stability of the carbon pool via reduction of the respiration costs, and (iii) allowing understory species to persist in situ, thereby preventing thermophilization of plant communities. We argue that while such a decoupling effect cannot wholly isolate understory climatic conditions from regional macroclimate, it has the potential to partly offset the climatic warming experienced in the Mediterranean forest understory due to anthropogenic climate change.

## Supplementary Information

Supplementary Information.

## Data Availability

The datasets used and/or analyzed during the current study are available from the corresponding author on reasonable request.
