# Supplementary material for: Topography modulates near-ground microclimate in the Mediterranean Fagus sylvatica treeline
Source: Sci Rep. 2021 Apr 14;11:8122. doi: 10.1038/s41598-021-87661-6 (PMC8046975; doi:10.1038/s41598-021-87661-6)
Supplement: Supplementary file 1 — Supplementary Information. [file 41598_2021_87661_MOESM1_ESM.docx]

Supplementary Material

Topography modulates near-ground microclimate in the Mediterranean *Fagus sylvatica* treeline

**Authors’ full names**

Angelo Rita^1,2,*^, Giuliano Bonanomi^1^, Emilia Allevato^1^, Marco Borghetti^2^, Gaspare Cesarano^1^, Valentina Mogavero^1^, Sergio Rossi^3,4^, Luigi Saulino^1^, Maurizio Zotti^1^, Antonio Saracino^1^

**Affiliations**

*^1^Dipartimento di Agraria, Università di Napoli Federico II, via Università 100, IT-80055 Portici (Napoli), Italy*

*^2^Scuola di Scienze Agrarie, Forestali, Alimentari e Ambientali, Università della Basilicata, Viale dell'Ateneo Lucano 10, IT-85100 Potenza, Italy*

*^3^Département des Sciences Fondamentales, Université du Québec à Chicoutimi, boulevard de l’Université Chicoutimi (Québec), Canada*

*^4^Key Laboratory of Vegetation Restoration and Management of Degraded Ecosystems, Guangdong Provincial Key Laboratory of Applied Botany, South China Botanical Garden, Chinese Academy of Sciences, Guangzhou, China*

**Corresponding author’s full address***

Name: Angelo Rita

E-mail: angelo.rita@unina.it

**Table S1.** Summary statistics of near-ground air temperature performed below-canopy treeline and in the open-field at north-west and south expositions. Min., minimum value; Max., maximum value; IQR, interquartile range; cv, coefficient of variation. In parenthesis the soil temperature. Tms G.S. is the growing season spanning from May to end of September, and defined by air temperature (Walter and Linderholm^1^) for the growing season start (first 5-day spell with daily mean temperatures > 5 °C) and the growing season end (10-day spell with temperatures < 5 °C). Ts is the growing season where time-span is reported in the table.

|  | North-West | North-West | South | South |
| --- | --- | --- | --- | --- |
|  | below-canopy | open-field | below-canopy | open-field |
|  | Tms G.S. | | | |
| Min. | 1.09 (4.2) | 1.43 (4.39) | 3.57 (6.02) | 4.7 (7.36) |
| Median | 10.96 (9.69) | 11.95 (11.88) | 13.67 (11.65) | 15.63 (15.4) |
| Mean | 10.46 (9.22) | 11.51 (11.17) | 13.17 (11.18) | 15.32 (15.19) |
| Max. | 17.86 (12.18) | 18.18 (16.03) | 20.29 (14.07) | 23.35 (21.17) |
| IQR | 5.72 (2.87) | 6.11 (4.1) | 6.74 (3.17) | 7.3 (5.16) |
| cv | 0.36 (0.22) | 0.33 (0.25) | 0.31 (0.19) | 0.29 (0.22) |
|  |  |  |  |  |
|  | Ts G.S. | | | |
| Duration | 25/05 - 08/11 | 25/05 - 09/11 | 17/05 - 28/11 | 10/05 - 28/11 |
| Min. | 0.13 (4.21) | 0.4 (3.7) | 0.3 (4.34) | 0.3 (4.34) |
| Median | 10.3 (9.11) | 11.24 (10.44) | 11.09 (10.75) | 11.04 (10.66) |
| Mean | 9.88 (8.92) | 10.79 (10.6) | 11.61 (10.5) | 11.47 (10.38) |
| Max. | 17.86 (12.18) | 18.18 (16.03) | 20.29 (14.07) | 20.29 (14.07) |
| IQR | 6.19 (3.29) | 6.3 (4.76) | 7.92 (3.6) | 7.91 (3.84) |
| cv | 0.39 (0.23) | 0.37 (0.28) | 0.41 (0.23) | 0.42 (0.23) |

### **Table S2**. Results of null random-slope models testing the effect of the temperature offset for near-ground average temperatures in the two expositions. Est, estimates; CI, confidence interval; p, P-value; sigma-squared (σ^2^), within-group variance; tau-zero-zero (τ_00_), between-group-variance; N, number of observations; ICC, Intra Class Correlation; R²m, marginal R²-values (fixed effects only); R²c, conditional R² values (fixed and random effects); G.S., growing season. The basic model is reported according to the Wilkinson-Rogers notation as T_offset_ ~ 1 + (1 | month/day/hour), where T_offset_ represents the near-ground temperature offset for South and North-West aspects in June-July-August (JJA) and December-January-February (DJF), and during the growing season (G.S., *i.e.*, from May to September).

|  | **South - JJA** | | | **South - DJF** | | | **North-West - JJA** | | | **North-West - DJF** | | | **South - G.S.** | | | **North-West - G.S.** | | |
| --- | --- | --- | --- | --- | --- | --- | --- | --- | --- | --- | --- | --- | --- | --- | --- | --- | --- | --- |
|  | *Est.* | *CI* | *p* | *Est.* | *CI* | *p* | *Est.* | *CI* | *p* | *Est.* | *CI* | *p* | *Est.* | *CI* | *p* | *Est.* | *CI* | *p* |
| (Intercept) | -2.6 | -2.82  –  -2.35 | **<0.001** | 0 | -0.72  –  0.74 | 0.986 | -1.2 | -1.45  –  -0.91 | **<0.001** | -0.1 | -6.47  –  6.32 | 0.981 | -2.4 | -2.68 –  -2.14 | **<0.001** | -1 | -1.39  –  -0.63 | **<0.001** |
| **Random**  **Effects** |  |  |  |  |  |  |  |  |  |  |  |  |  |  |  |  |  |  |
| σ^2^ | 0.35 | | | 0.12 | | | 0.25 | | | 0.03 | | | 0.31 | | | 0.14 | | |
| τ_00_ | 14.85  hour:(day:month) | | | 1.89  hour:(day:month) | | | 5.61  hour:(day:month) | | | 0.48  hour:(day:month) | | | 13.35  hour:(day:month) | | | 4.27  hour:(day:month) | | |
|  | 0.03 day:month | | | 0.99 day:month | | | 0.08 day:month | | | 1.90 day:month | | | 0.10 day:month | | | 0.14 day:month | | |
|  | 0.02 month | | | 0.38 month | | | 0.05 month | | | 31.89 month | | | 0.07 month | | | 0.18 month | | |
| ICC | 0.98 | | | 0.97 | | | 0.96 | | | 1 | | | 0.98 | | | 0.97 | | |
| N | 24 hour | | | 24 hour | | | 24 hour | | | 24 hour | | | 24 hour | | | 24 hour | | |
|  | 31 day | | | 31 day | | | 31 day | | | 31 day | | | 31 day | | | 31 day | | |
|  | 3 month | | | 3 month | | | 3 month | | | 3 month | | | 5 month | | | 5 month | | |
| Obs. | 4002 | | | 4320 | | | 4445 | | | 4320 | | | 6306 | | | 6720 | | |
| R^2^m/R^2^c | 0.000 / 0.977 | | | 0.000 / 0.965 | | | 0.000 / 0.957 | | | 0.000 / 0.999 | | | 0.000 / 0.978 | | | 0.000 / 0.971 | | |

**Table S3.** Results of general Additive Mixed Models (GAMMs) for south (above) and north-west (below) treelines. Section A reports parametric coefficients, and section B report the smooth terms (*s*). Est, estimates; Std. Error, Standard error of the estimates; p, P-value. The basic model is reported according to the Wilkinson-Rogers notation as T_offset_ ~ *s*(T_open-field_) + *sin*(hour) + *cos*(hour), random = list(day.month= ~ 1) for the nonparametric GAM and as T_offset_ ~T_open-field_ + *sin*(hour) + *cos*(hour) + (1 | day.month) for the parametric LMM, where T_offset_ represents the near-ground temperature offset for South and North-West aspects during the growing season (i.e., from May to September), *s* denotes the nonparametric smoothing term, T_open-field_ represents the open-field near-ground temperature, and day.month is the combined random term.

| Summary of South |  |  |  |  |
| --- | --- | --- | --- | --- |
| A. parametric coefficients | Est. | Std. Error | t-value | p |
| (Intercept) | -2.4131 | 0.1696 | -14.2293 | *< 0.0001* |
| *sin*(hour) | 0.4209 | 0.0216 | 19.4836 | *< 0.0001* |
| *cos*(hour) | 0.1182 | 0.0248 | 4.7714 | *< 0.0001* |
| B. smooth terms | edf | Ref.df | F-value | p-value |
| *s*(T_open-field_) | 7.9443 | 7.9443 | 3939.6017 | *< 0.0001* |
|  |  |  |  |  |
| Summary of North-West |  |  |  |  |
| A. parametric coefficients | Est. | Std. Error | t-value | p |
| (Intercept) | -2.4131 | 0.1696 | -14.2293 | *< 0.0001* |
| *sin*(hour) | 0.4209 | 0.0216 | 19.4836 | *< 0.0001* |
| *cos*(hour) | 0.1182 | 0.0248 | 4.7714 | *< 0.0001* |
| B. smooth terms | edf | Ref.df | F-value | p-value |
| *s*(T_open-field_) | 7.9443 | 7.9443 | 3939.6017 | *< 0.0001* |

**Table S4.** Results from linear mixed-effects models predicting understory near-ground temperature as a function of open-field temperatures and daytime (as sin and cos of hours) as predictors and month and day as a nested random effect to take into account the non-independence of data. Est, estimates; CI, confidence interval; p, P-value; sigma-squared (σ^2^), within-group variance; tau-zero-zero (τ_00_), between-group-variance; N, number of observations; ICC, Intra Class Correlation; R²m, marginal R²-values (fixed effects only); R²c, conditional R² values (fixed and random effects). The basic model is reported according to the Wilkinson-Rogers notation as T_understory_ ~T_open-field_ + sin(hour) + cos(hour) + (1 | month/day), where T_understory_ represents the near-ground understory temperature, T_open-field_ represents the open-field near-ground temperature during the growing season (i.e., from May to September), and month/day denoted the nested random term.

|  | **South** | | | **North-West** | | |
| --- | --- | --- | --- | --- | --- | --- |
| *Predictors* | *Estimates* | *CI* | *p* | *Estimates* | *CI* | *p* |
| (Intercept) | 3.65 | 3.54 – 3.76 | **<0.001** | 1.71 | 1.63 – 1.80 | **<0.001** |
| T_open-field_ | 0.6 | 0.60 – 0.61 | **<0.001** | 0.76 | 0.76 – 0.77 | **<0.001** |
| sin(hour) | 0.3 | 0.26 – 0.34 | **<0.001** | 0.09 | 0.04 – 0.15 | **0.001** |
| cos(hour) | 0.01 | -0.04 – 0.06 | 0.641 | -0.51 | -0.55 – -0.47 | **<0.001** |
| **Random Effects** | | | | | | |
| σ^2^ | 0.66 | | | 1.03 | | |
| τ_00_ | 3.04 day:month | | | 1.78 day:month | | |
|  | 1.78 month | | | 0.51 month | | |
| ICC | 0.88 | | | 0.69 | | |
| N | 31 day | | | 31 day | | |
|  | 5 month | | | 5 month | | |
| Obs. | 6306 | | | 6720 | | |
| R^2^m/R^2^c | 0.718 / 0.966 | | | 0.820 / 0.944 | | |

**Table. S5**. Pairs of correlations among microclimatic measured variables (T, temperature; M, moisture) in the open-field and below the canopy in the north-western slope.

|  | Soil T_below-canopy_ | Soil T_open-field_ | Soil M_below-canopy_ | Soil M_open-field_ | Air T_below-canopy_ |
| --- | --- | --- | --- | --- | --- |
| Soil T_open-field_ | 0.96 |  |  |  |  |
| Soil M_below-canopy_ | -0.24 | -0.18 |  |  |  |
| Soil M_open-field_ | 0.28 | 0.29 | 0.72 |  |  |
| Air T_below-canopy_ | 0.84 | 0.88 | -0.19 | 0.22 |  |
| Air T_open-field_ | 0.78 | 0.88 | -0.13 | 0.24 | 0.94 |

**Table. S6**. Pairs of correlations among microclimatic measured variables (T, temperature; M, moisture) in the open-field and below the canopy in the southern slope.

|  | Soil T_below-canopy_ | Soil T_open-field_ | Soil M_below-canopy_ | Soil M_open-field_ | Air T_below-canopy_ |
| --- | --- | --- | --- | --- | --- |
| Soil T_open-field_ | 0.96 |  |  |  |  |
| Soil M_below-canopy_ | -0.7 | -0.71 |  |  |  |
| Soil M_open-field_ | -0.48 | -0.45 | 0.81 |  |  |
| Air T_below-canopy_ | 0.85 | 0.9 | -0.65 | -0.42 |  |
| Air T_open-field_ | 0.77 | 0.85 | -0.58 | -0.36 | 0.96 |

**Supplementary Method**

Metrics of land surface phenology and greenness are derived from NDVI time series extracted from MOD13Q1 250m spatial resolution and 16-day temporal resolution for the period 2016-2019 via *rgee*^2^+*GEE* bridge packages in R statistical environment. The output (see figures below) was processed using the *greenbrown*^3^ package version 2.2 in R environment to calculate the phenology metrics on time series from the start of the season (SOS), end of the season (EOS), and length of the season (LOS), spanning from 2009 to 2019. A description of these metrics and a discussion on the caveats and challenges of phenology detection from remote sensing-derived vegetation index time series can be found in Forkel et al.^3^. We fitted a double logistic curve to observed values using the function as described in Elmore et al.^4^.

Monthly normalized difference snow index (NDSI) values was also calculated using Landsat 4, 5, 7, 8 SR with 30m spatial resolution for the period 1984-2020 as the ratio of the difference in VIS and SWIR reflectance; NDSI = ((band 4-band 6) / (band 4 + band 6)). A pixel with NDSI > 0.0 is considered to have some snow present. A pixel with NDSI <= 0.0 is a snow-free land surface.

**Fig. S1**. Maximum (red) and minimum (blue) median monthly near-ground temperature (°C) distributions in the high elevation open-field north-western (upper panels) and south aspects (lower panels), respectively. Each box represents the 75^th^ to 25^th^ percentiles, and the line inside the median; upper and lower marks are the largest to smallest observation values, which are less than or equal to the upper and lower quartile plus 1.5 the length of the interquartile range; circles outside the lower-upper mark range are outliers.

**
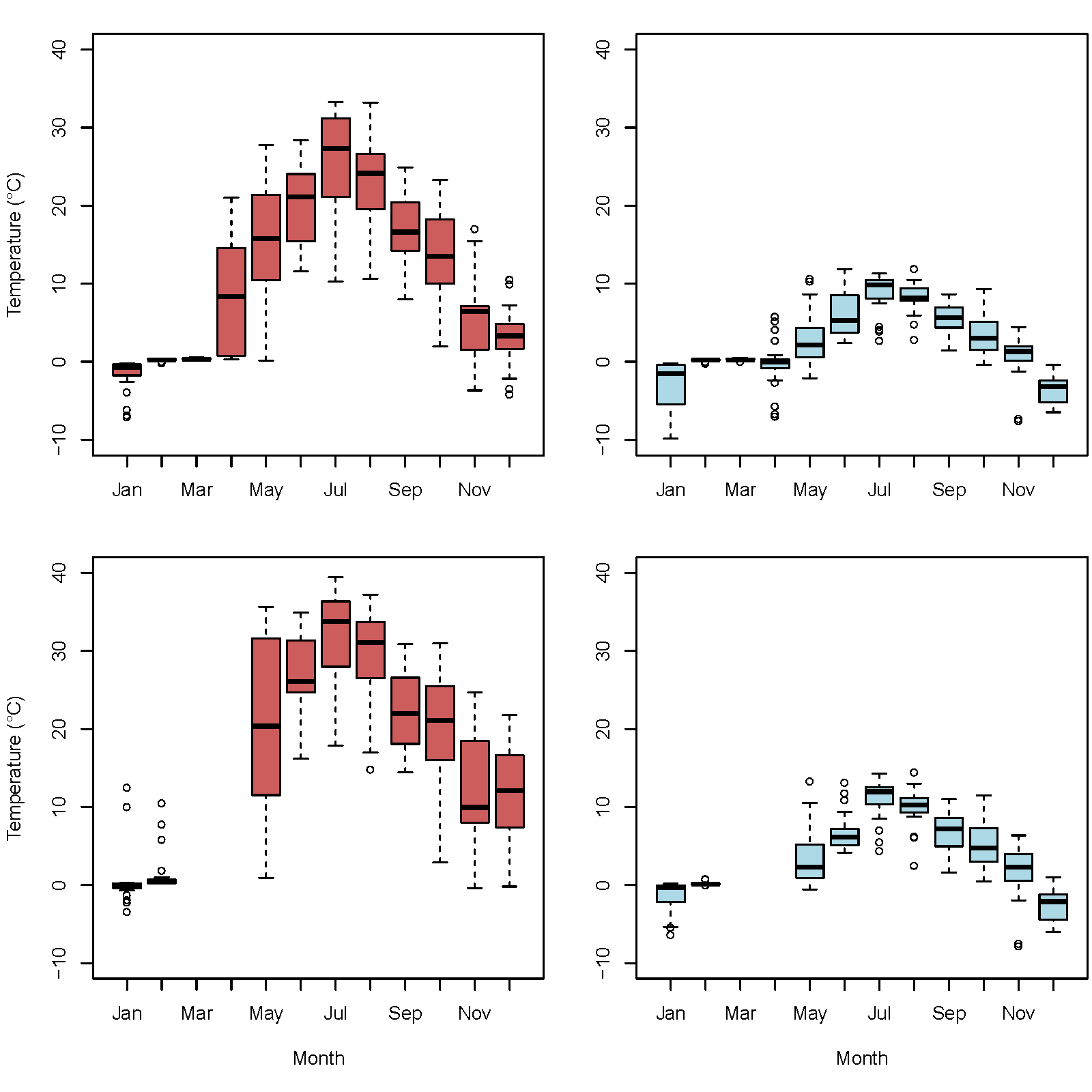
**

**Fig. S2**. Average daily near-ground air temperature (°C) recorded in the open-field (blue) and below the forest treeline canopy (red) at north-west (top) and south (bottom) aspects, respectively.

**
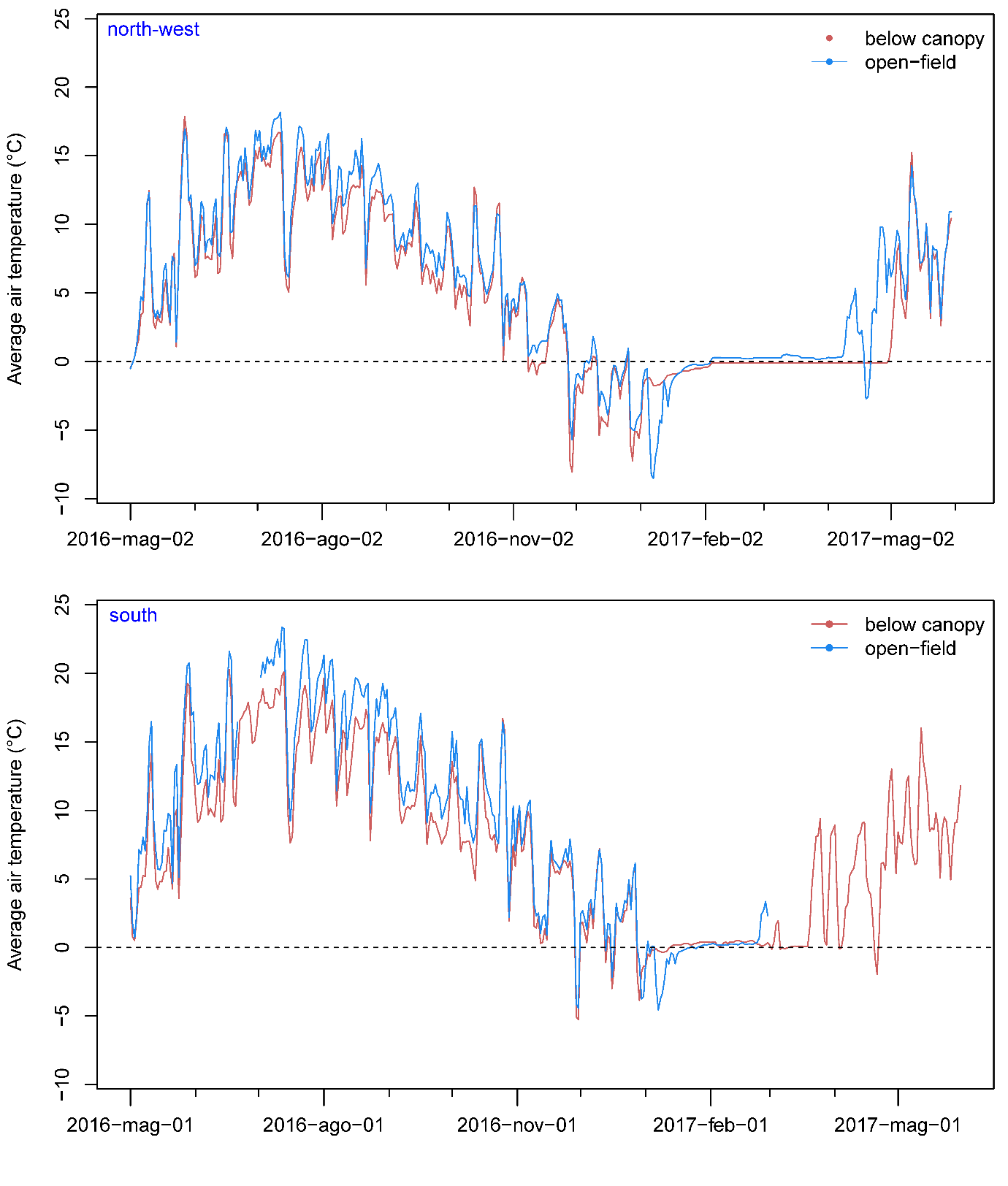
**

**Fig. S3**. Near-ground air temperature offset values for maximum temperatures during summer (JJA), and winter (DJF). Maximum temperatures are consistently cooler under-canopy (microclimate) compared to open field (macroclimate). Temperature offsets (mean ± s.e.) are based on mixed-effects models with months, days, and hours as nested random-effect terms (the full statistics are reported in Supplementary Material Table S2). Y-axis scales are not fixed.


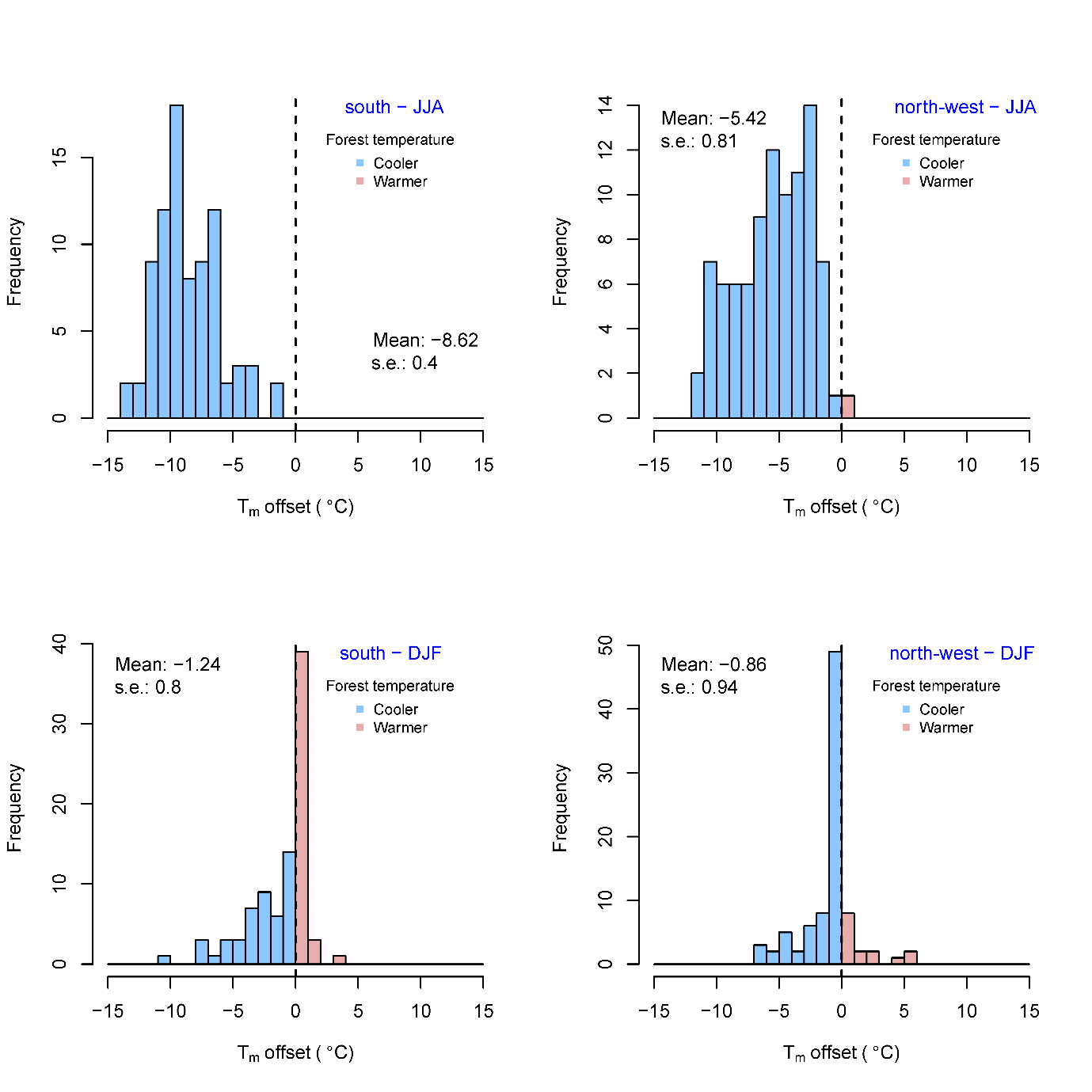


**Fig. S4**. Near-ground air temperature offset values for minimum temperatures during summer (JJA), and winter (DJF). Maximum temperatures are consistently cooler under-canopy (microclimate) compared to open field (macroclimate). Temperature offsets (mean ± s.e.) are based on mixed-effects models with months, days, and hours as nested random-effect terms (the full statistics are reported in Supplementary Material Table S2). Y-axis scales are not fixed.


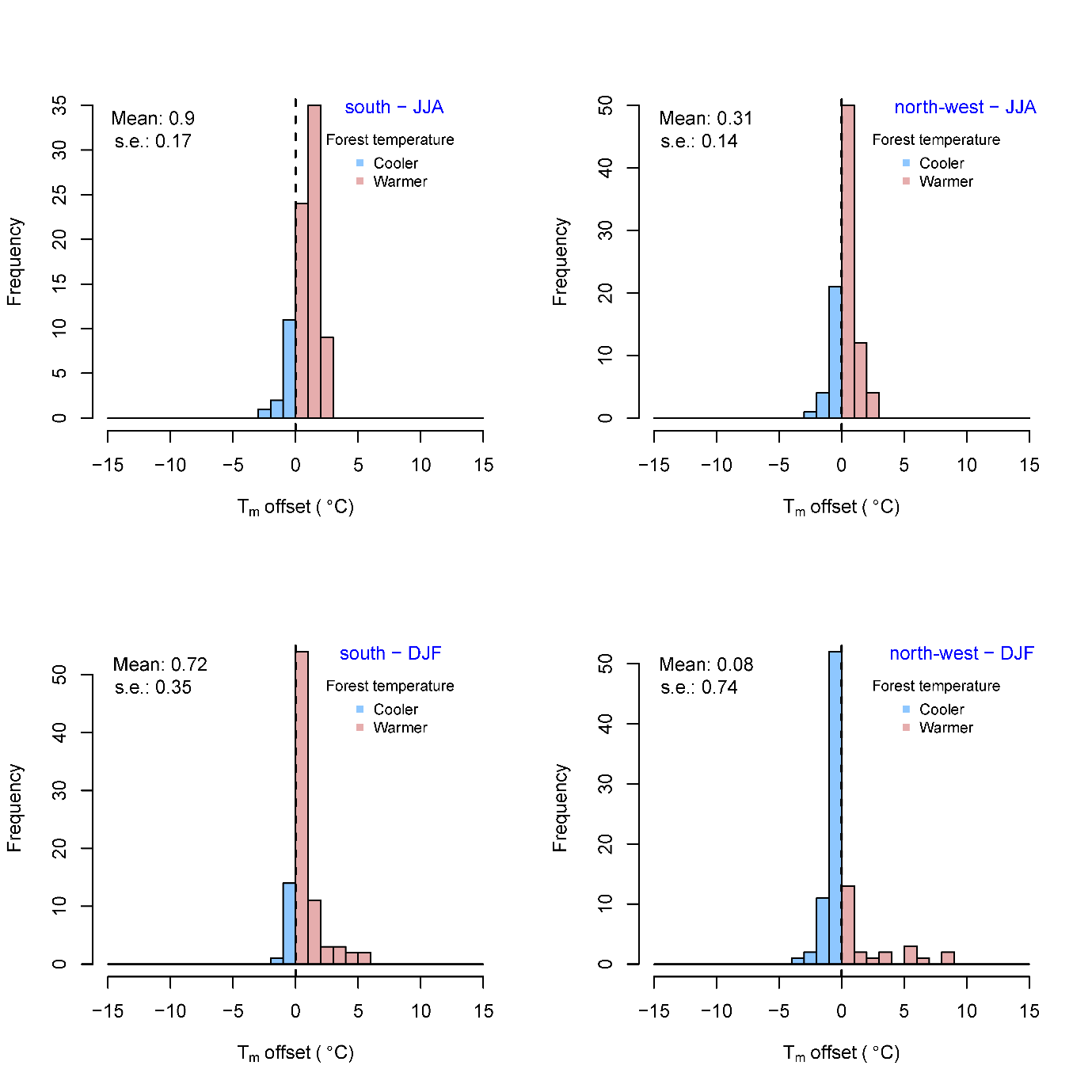


**Fig. S5**. Daytime patterns in near-ground air temperature for a selected random day for each season (from left to right: spring, summer, autumn, winter). The *F. sylvatica* treelines on the northern and southern slopes are shown on top and bottom panels, respectively. Blue and red lines represent the near-ground surface temperature below the canopy and in the open field, respectively.


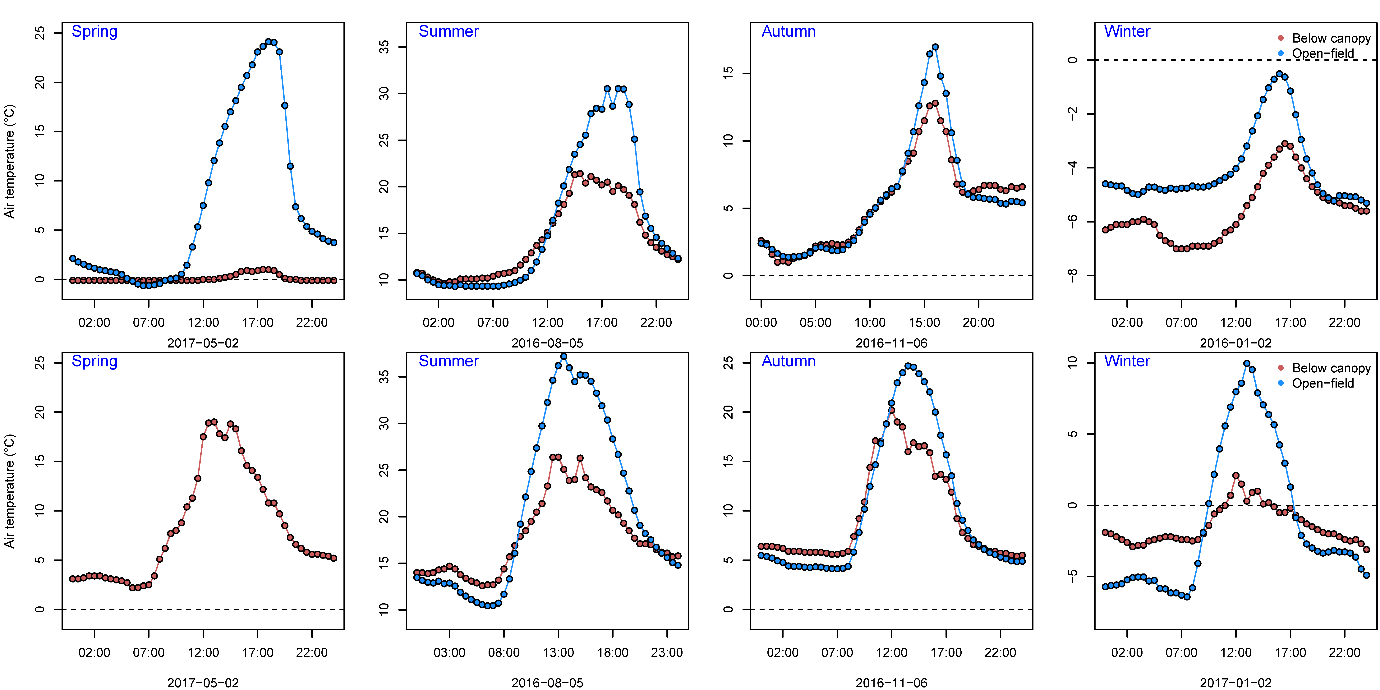


**Fig. S6**. Average daily soil temperature (°C) recorded in the open-field (blue) and below the treeline forest canopy (red) at north-west (top) and south (bottom) aspects, respectively.

**
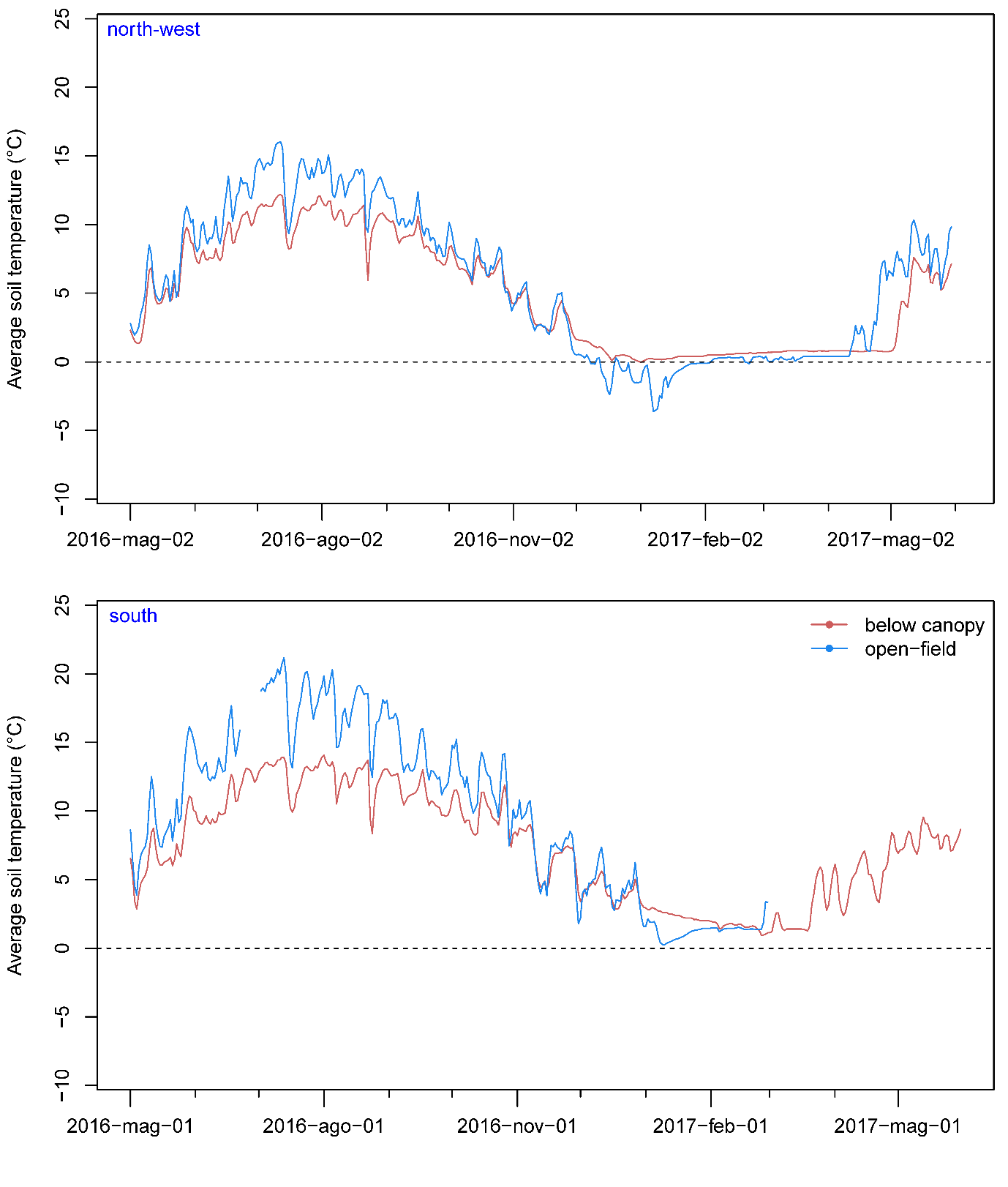
**

**Fig. S7**. Normalized difference snow index (NDSI) values calculated as the ratio of the difference in VIS and SWIR reflectance; NDSI = ((band 4-band 6) / (band 4 + band 6)). A pixel with NDSI > 0.0 is considered to have some snow present. A pixel with NDSI <= 0.0 is a snow-free land surface.


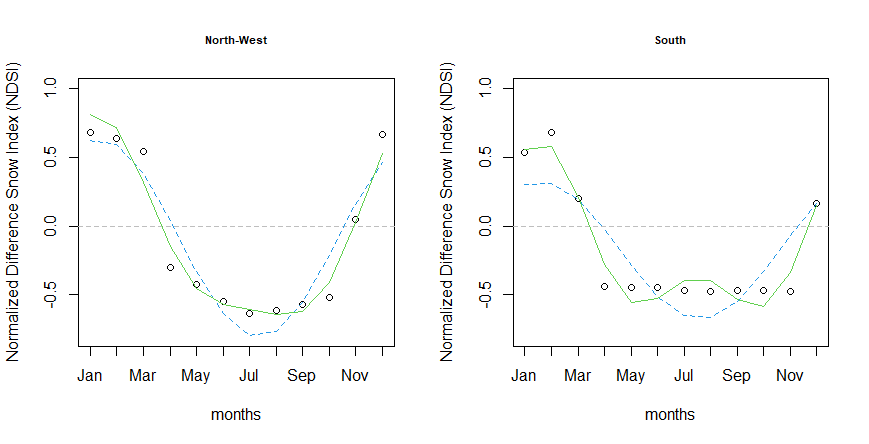


**Fig. S8**. Average daily soil moisture (m^3^/m^3^) recorded in the open-field (blue) and below the forest treeline canopy (red) at north-west (top) and south (bottom) aspects, respectively.

**
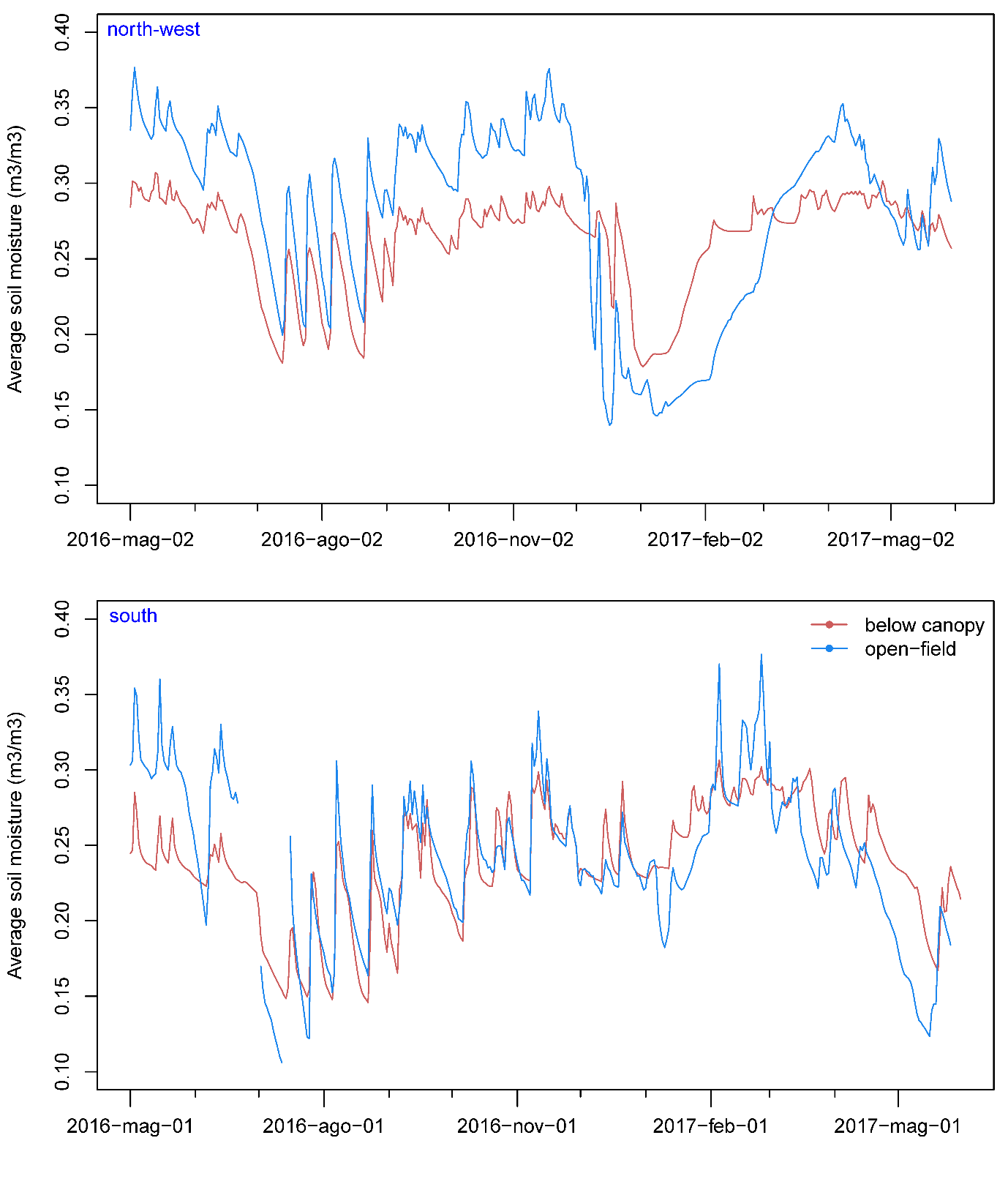
**

**Fig. S9**. Daily trend of direct solar irradiance (W m-2) receipt from North (blue) and South (red) facing slopes on Serra del Prete Mountain (Pollino, Italy). Length of growing season differs according to the aspect of the surface (azimuth): 15 May to 15 September for N-facing slope and 15 April to 15 October for S-facing slope.


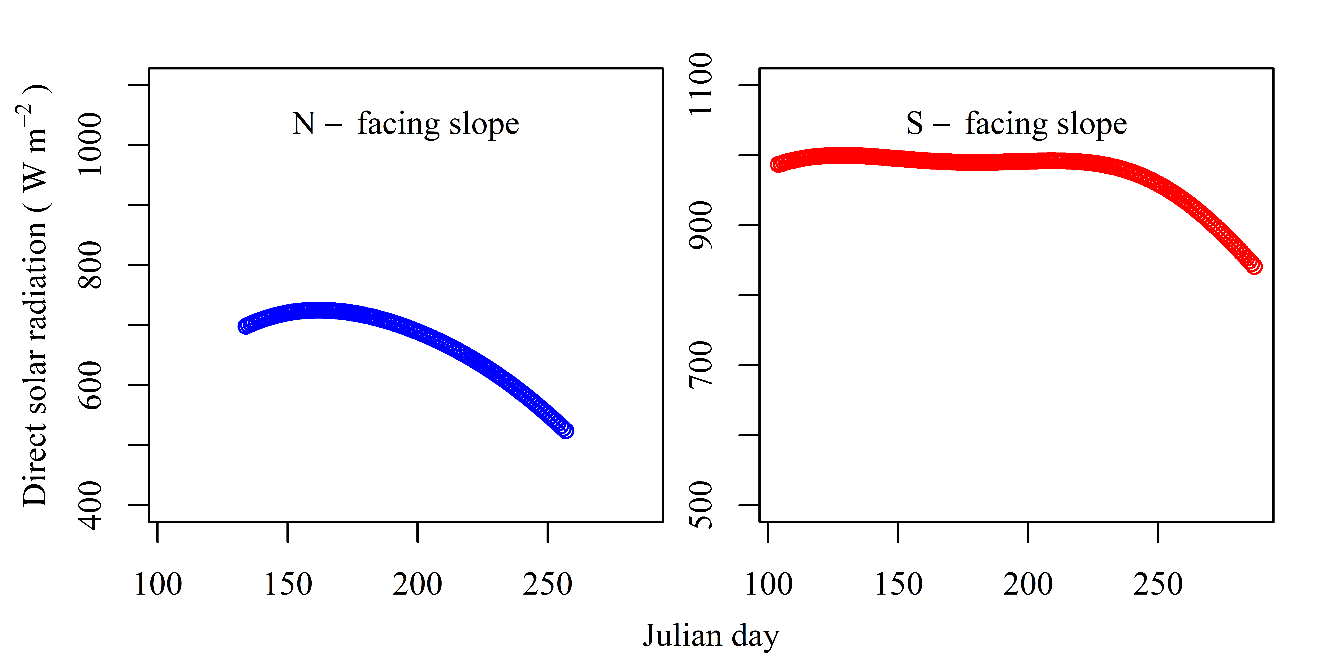


References:

1. Walther, A., Linderholm, H.W. A comparison of growing season indices for the Greater Baltic Area. *Int. J. Biometeorol*. **51**,107–118 (2006)
2. Aybar, C., Wu, Q., Bautista, L., Yali, R., & Barja, A. rgee: An R package for interacting with Google Earth Engine. *Journal of Open Source Software* **5**, 2272 (2020).
3. Forkel, M., Migliavacca, M., Thonicke, K., Reichstein, M., Schaphoff, S., Weber, U., Carvalhais, N.. [Codominant water control on global interannual variability and trends in land surface phenology and greenness.](http://onlinelibrary.wiley.com/doi/10.1111/gcb.12950/abstract) *Glob. Change. Biol*. **21**, 3414–3435 (2015).
4. Elmore, A.J., S.M. Guinn, B.J. Minsley and A.D. Richardson: Landscape controls on the timing of spring, autumn, and growing season length in mid-Atlantic forests. - *Glob. Change. Biol.* **18**, 656-674 (2012).
